# Predicting the onset of freezing of gait in Parkinson’s disease

**DOI:** 10.1186/s12883-022-02713-2

**Published:** 2022-06-07

**Authors:** Fengting Wang, Yixin Pan, Miao Zhang, Kejia Hu

**Affiliations:** 1 Department of Neurosurgery, Ruijin Hospital, Shanghai Jiao Tong University School of Medicine, Shanghai, 200025 China; 2 Center for Functional Neurosurgery, Ruijin Hospital, Shanghai Jiao Tong University School of Medicine, Shanghai, 200025 China; 3 School of Medicine, Shanghai Jiao Tong University, Shanghai, 200025 China; 4 Department of Nuclear Medicine, Ruijin Hospital, Shanghai Jiao Tong University School of Medicine, Shanghai, 200025 China; 5 Laboratory of Digital Medicine, Wuxi People’s Hospital, Nanjing Medical University, Wuxi, 214023 China

**Keywords:** Freezing of gait, Risk factors, Parkinson's disease, Prediction

## Abstract

**Background:**

Freezing of gait is a debilitating symptom of Parkinson’s disease associated with high risks of falls and poor quality of life. While productive therapy for FoG is still underway, early prediction of FoG could help high-risk PD patients to take preventive measures. In this study, we predicted the onset of FoG in *de novo* PD patients using a battery of risk factors from patients enrolled in PPMI cohort.

**Methods:**

Baseline characteristics were compared between subjects who developed FoG (68 patients, 37.2%, pre-FoG group) during the five-year follow up and subjects who did not (115 patients, 62.8%, non-FoG group). A multivariate logistic regression model was built based on backward stepwise selection of factors that were associated with FoG onset in the univariate analysis. ROC curves were used to assess sensitivity and specificity of the predictive model.

**Results:**

At baseline, age, PIGD score, cognitive functions, autonomic functions, sleep behavior, fatigue and striatal DAT uptake were significantly different in the pre-FoG group relative to the non-FoG group. However, there was no difference in genetic characteristics between the two patient sets. Univariate analysis showed several motor and non-motor factors that correlated with FoG, including PIGD score, MDS-UPDRS part II score, SDMT score, HVLT Immediate/Total Recall, MOCA, Epworth Sleepiness Scale, fatigue, SCOPA-AUT gastrointestinal score, SCOPA-AUT urinary score and CSF biomarker Abeta_42_. Multivariate logistic analysis stressed that high PIGD score, fatigue, worse SDMT performance and low levels of Abeta_42_ were independent risk factors for FoG onset in PD patients.

**Conclusions:**

Combining motor and non-motor features including PIGD score, poor cognitive functions and CSF Abeta can identify PD patients with high risk of FoG onset.

**Supplementary Information:**

The online version contains supplementary material available at 10.1186/s12883-022-02713-2.

## Background

Freezing of gait (FoG) refers to a sudden inability to initiate or continue gait [1]. As a common symptom with increasing frequency as Parkinson’s disease (PD) progresses, it has a significant impact on the patient quality of life [1]. FoG is hard to study due to its transient occurrence and multifaceted pathophysiology. The locomotor network, especially the subthalamic nucleus (STN), globus pallidus internus (GPi), and substantia nigra pars reticulata (SNr) can all contribute to the movement breakdown [2]. Moreover, cognitive and limbic networks are also involved in this gait abnormality [3]. While effective therapies for FoG are still under investigation, early prediction of FoG may identify future patients for preventive management.

Several methods are adopted to predict FoG onset [4]. Wearable sensors, which can objectively detect the gait disturbance, are accessible to a limited number of patients [5]. Clinical variables and neuro-physiological biomarkers, however, are more common in clinical use. Various associations between FoG and clinical observations, genetic variations, Cerebrospinal fluid (CSF) biomarkers as well as neuro-imaging features have been discovered [6–14]. Motor factors such as rigidity, postural instability, and bradykinesia are predictive of FoG [6]. Non-motor factors, including specific cognitive deficits, mood disorders, and autonomic dysfunctions, are also associated with FoG occurrence [7–10]. CSF biomarkers such as β-amyloid 1–42 (Abeta_42_) and gene mutations including APOE ε4 have been associated with the symptom onset [8]. Striatal dopaminergic denervation, which can be examined using dopamine transporter (DAT) scans and single photon emission computed tomography (SPECT) imaging, is also associated with FoG pathology [15]. Although past studies have identified several FoG risk factors, few have combined these factors to predict FoG onset.

Instead of focusing on one aspect of clinical or imaging assessments, our study analyzed a comprehensive battery of indicators. Motor and non-motor factors, genetic characteristics, CSF biomarkers as well as the neuroimaging parameters were evaluated using longitudinal data of five-year visit from the PPMI cohort. Our goal was to determine the early symptoms and characteristics exhibited in PD patients before FoG occurrence.

## Methods

### Study design and participants

Data underlying this study were obtained from the Parkinson’s Progression Markers Initiative (PPMI) database (www.ppmi-info.org/data). PPMI is a comprehensive observational, multi-center study designed to identify biomarkers in participants with early untreated (*de novo*) PD at enrollment. The inclusion criteria for enrollment into PPMI were: a) age: > 30 years old, b) untreated with PD medications, c) PD diagnosed within two years, d) Hoehn and Yahr < 3, and e) patients with either at least two of resting tremor, bradykinesia, or rigidity (must have either resting tremor or bradykinesia) or a single asymmetric resting tremor or asymmetric bradykinesia at enrollment. Since PD diagnosis is based on the presence of bradykinesia, only patients that exhibited bradykinesia, with or without resting tremor or rigidity were involved in our study [16]. The database was accessed on September 10, 2021.

Here, pre-FoG and non-FoG patient groups were defined based on the presence or absence of FoG during the 5-year follow up. FoG was assessed using MDS-UPDRS (Movement Disorders Society- Unified Parkinson’s Disease Rating Scale) Part II item ‘freezing’ as well as Part III item ‘freezing of gait’. MDS-UPDRS Part II assessed motor experiences of daily living and was included in the questionnaire completed by participants at each follow-up visit. MDS-UPDRS Part III assessed the motor signs of PD and was administered by the investigator. Subjects who had started PD medication (levodopa or dopamine agonists) would have an annual assessment of the motor exam (Part III) in a practically defined off state and these assessments would be repeated one hour after receiving medication at clinic. Patients scoring above zero for either of the items at any point during the follow-up visit were considered as having FoG.

### Baseline assessments

Various clinical variables, including imaging assessments and genetic patterns, were recorded at baseline. Motor indicators included resting tremor, rigidity, bradykinesia, TD/PIGD classification, PIGD score, tremor score and MDS‐UPDRS part II and III. Modified Schwab & England ADL score was used to evaluate activities of daily living. Non-motor indicators included: MDS-UPDRS part I to assess non-motor experiences of daily living; MOCA (Montreal Cognitive Assessment) to assess global cognition; MCI test scores to evaluate test-based mild cognitive impairment (MCI); HVLT (Hopkins Verbal Learning Test) to assess memory; the 40‐item UPSIT (University of Pennsylvania Smell Identification Test) to assess olfactory function; Benton Judgement of Line Orientation Test to assess visuospatial function; Epworth Sleepiness Scale and REM sleepbehavior disorder questionnaire to assess sleep behavior; Geriatric Depression Scale, STAI (State-Trait Anxiety Inventory), and QUIP (Questionnaire for Impulsive‐Compulsive Disorders) to assess neuron-behavior; SDMT (Symbol Digit Modalities Test) to assess attention and processing speed; Letter Number Sequencing and semantic (animal) fluency test to assess executive abilities-working memory; SCOPA‐AUT (Scales for Outcomes in Parkinson's Disease‐Autonomic) to assess autonomic functions.

TD/PIGD classification is defined by Tremor score/PIGD score. Tremor score is the mean of the following variables from MDS-UPDRS items: 2.10, 3.15a, 3.15b, 3.16a, 3.16b, 3.17a, 3.17b, 3.17c, 3.17d, 3.17e, 3.18. PIGD score is the mean of the following variables from MDS-UPDRS items: 2.12, 2.13, 3.10, 3.11, and 3.12. If ratio ≥ 1.15, or if PIGD score = 0 and Tremor score > 0, then subject is TD. If ratio ≤ 0.9 then subject is PIGD. If ratio > 0.9 and < 1.15, or if Tremor score and PIGD score = 0, then subject is indeterminate.

CSF was collected using standardized lumbar puncture procedures. Its shipment and storage were conducted as described in the PPMI biologics manual (ppmi-info.org/study-design). CSF biomarkers amyloid-β1-42 (Abeta), total tau (Tau), and phosphorylated tau (pTau) were analyzed using the xMAP-Luminex platform with INNOBIA AlzBio3 immunoassay kit-based reagents (Fujirebio-Innogenetics, Ghent, Belgium). CSF α-synuclein was analyzed using ELISA kit (Covance, Dedham, MA).

DNA of the participants was extracted from whole blood using the study protocol described in the PPMI biologics manual (ppmi-info.org/study-design). Genetic patterns of MAPT (microtubule-associated protein tau), APOE ε4 (the apolipoprotein ε4) allele, mutations in SNCA (α-synuclein) including SNCA_rs3910105 and SNCA_rs356181 were evaluated as described in previous studies [17].

Indexes of reconstructed and attenuation-corrected 123I-FP-CIT SPECT imaging data were downloaded from PPMI. All participants underwent DAT imaging to measure the amount of dopamine in the brain using SPECT with 123I-ioflupane as DAT tracer. Imaging was done on a Siemens or General Electric SPECT tomograph, 3–4 h after 123I-FP-CIT injection. The standard procedures for CSF biomarkers examinations, genotyping and DAT SPECT imaging were described before [17]. Subjects with missing data were excluded from the study.

### Statistical analysis

Statistical analyses were done on R v.4.0.1 (R foundation for Statistical Computing, Vienna, Austria) and SPSS 18.0 (IBM). Normally distributed continuous data were examined by Shapiro-Wilks test and presented as mean (standard deviation). Non-normally distributed continuous data were presented as median [quartile]. Student’s t-test, Kruskal Willis test, Chi square and fisher exact test were used to compare baseline features in the pre-FoG and non-FoG groups. *P* < 0.05 was presented with ‘*’. The evolution of MDS-UPDRS scores was calculated by subtracting the MDS-UPDRS scores at each annual visit from baseline MDS-UPDRS scores. Binary logistic regression was used to identify potential risk factors for FoG onset. For multivariate analysis, a logistic regression model was built based on a backward stepwise selection with the significance level at which variables were entered and removed from the model as *p* = 0.05. If variables were highly related (r > 0.5), the variable with the lower *p* value was entered as an independent variable. To exclude covariates, we adjusted our multivariate logistic model for age, disease duration, and gender. Odds ratio (OR) and 95% CI were reported for bivariate and multivariate analyses. Receiver operating characteristic (ROC) curves were used to assess sensitivity and specificity of the predictive model. The Hosmer and Lemeshow goodness-of-fit test was used to assess the model calibration.

## Results

### Baseline characteristics

Of the 423 patients included in the study, 348 patients exhibited bradykinesia at baseline. Of these, 23 patients developed FoG at baseline, 74 patients had no visit data on year five and 68 patients had missing data at baseline visit. A total of 183 patients were finally involved in the study. During the 5-year follow-up, 68 (37.2%) of 183 PD patients developed FoG. The cumulative incidence of FoG was 12.0%, 19.7%, 23.5%, 31.1% and 37.2% at 1-, 2-, 3-, 4- and 5-year follow-up (Table S1). Among these patients, 31.1% of patients (57/183) reported ‘freezing when walking’ in their activities of daily living, while 17.5% of patients (32/183) were defined as FoG by the investigator in clinic (Table S1, Figure S1). Patients who developed FoG within 5 years (pre-FoG patients) differed significantly from those who did not (non-FoG patients) with regard to age, age at symptom onset and striatal DAT uptake. However, no significant difference was observed in disease duration, genetic characteristics, the side most affected at onset, and CSF biomarkers at baseline (Table 1).

**Table 1:** Demographic, disease, imaging and genetic characteristics of pre-FoG and non-FoG subjects at baseline

	**Non-FoG (*n* = 115)**	**Pre-FoG (*n* = 68)**	***P***
**Demographic information**			
Age (years)	60.3 [52.8;68.5]	64.9 [56.8;69.6]	0.019*
Age at Symptom Onset (years)	58.6 [50.4;66.3]	62.4 [55.3;68.3]	0.009*
Duration of Disease since Diagnosis (Months)	3.87 [2.33;7.22]	4.82 [2.46;7.02]	0.606
Gender, female	34 (29.6%)	19 (27.9%)	0.948
Years of education	16.0 [14.0;18.0]	16.0 [14.0;18.0]	0.848
Family members with PD (any)	27 (23.5%)	19 (27.9%)	0.620
**Disease characteristics**			
Side most affected at PD onset			0.368
Left	49 (42.6%)	33 (48.5%)	
Right	65 (56.5%)	33 (48.5%)	
Symmetric	1 (0.87%)	2 (2.94%)	
**SPECT-DAT**			
Mean caudate DAT uptake	2.13 (0.49)	1.90 (0.53)	0.005*
Mean putamen DAT uptake	0.86 [0.70;1.03]	0.74 [0.57;0.85]	0.001*
Mean striatum DAT uptake	1.50 (0.35)	1.33 (0.39)	0.003*
**CSF biomarkers**			
Abeta	900 [704;1290]	881 [622;1072]	0.088
Tau	165 [135;210]	159 [140;200]	0.659
pTau	13.6 [11.2;17.2]	13.9 [11.3;17.2]	0.901
aSyn	1462 [1121;1801]	1423 [1141;1724]	0.692
**Genetic Pattern**			
APOE			0.938
e2/e2	1 (0.87%)	0 (0.00%)	
e2/e4	4 (3.48%)	1 (1.47%)	
e3/e2	13 (11.3%)	10 (14.7%)	
e3/e3	68 (59.1%)	40 (58.8%)	
e4/e3	26 (22.6%)	16 (23.5%)	
e4/e4	3 (2.61%)	1 (1.47%)	
SNCA_rs356181			0.414
C/C	38 (33.0%)	20 (29.4%)	
C/T	48 (41.7%)	35 (51.5%)	
T/T	29 (25.2%)	13 (19.1%)	
SNCA_rs3910105			0.427
C/C	20 (17.4%)	8 (11.8%)	
C/T	60 (52.2%)	34 (50.0%)	
T/T	35 (30.4%)	26 (38.2%)	
APOE Genotype—number of e4 alleles	0.31 (0.52)	0.28 (0.48)	0.659
MAPT			1.000
H1/H1	77 (67.0%)	46 (67.6%)	
H1/H2	32 (27.8%)	19 (27.9%)	
H2/H2	6 (5.22%)	3 (4.41%)	

*P* <0.05 was presented with ‘*’

Among the motor and non-motor parameters of non-FoG and pre-FoG patients, significant difference was observed in motor indicators including PIGD score, TD/PIGD classification, MDS-UPDRS Part II score and non-motor indicators such as SDMT score, Epworth Sleepiness Scale score, HVLT Immediate/Total Recall, SCOPA-AUT Gastrointestinal score and MOCA score at baseline (Table 2). Relative to non-FoG patients, those in the pre-FoG cohort had a significant increase in MDS UPDRS scores at year five, indicating a severer disease progression within 5 years.

**Table 2:** Motor and non-motor assessments of pre-FoG and non-FoG subjects at baseline and their evolution of MDS-UPDRS scores

	**Non-FoG**	**Pre-FoG**	***P***
	**(*n* = 115)**	**(*n* = 68)**	
**Motor assessments**			
Categorical Hoehn & Yahr			0.505
Stage 1	54 (47.0%)	30 (44.1%)	
Stage 2	61 (53.0%)	37 (54.4%)	
Stages 3–5	0 (0.00%)	1 (1.47%)	
Total Rigidity Score	3.00 [2.00;6.00]	3.50 [2.00;5.00]	0.888
TD/PIGD classification			0.003*
TD	90 (78.3%)	37 (54.4%)	
PIGD	14 (12.2%)	19 (27.9%)	
Indeterminate	11 (9.57%)	12 (17.6%)	
PIGD score	2.00 [1.00;3.00]	3.00 [3.00;4.00]	< 0.001
Tremor Score	4.00 [2.00;6.00]	3.00 [1.75;5.00]	0.302
Modified Schwab & England ADL Score	95.0 [90.0;100]	90.0 [90.0;100]	0.106
MDS-UPDRS Part II Score	4.00 [2.00;7.00]	6.00 [3.00;9.25]	0.004*
MDS-UPDRS Part III Score	18.0 [14.0;24.5]	21.0 [15.8;25.0]	0.205
MDS-UPDRS Total Score	28.0 [21.0;36.0]	33.5 [25.8;41.0]	0.009*
Non-motor assessments			
MDS-UPDRS Part I Score	5.00 [2.00;6.00]	6.00 [2.75;7.00]	0.031*
MDS-UPDRS Part I Features of Dopamine Dysregulation Syndrome			0.629
0	113 (98.3%)	66 (97.1%)	
1	2 (1.74%)	2 (2.94%)	
MDS-UPDRS Part I Fatigue			< 0.001*
0	64 (55.7%)	31 (45.6%)	
1	49 (42.6%)	24 (35.3%)	
2	2 (1.74%)	9 (13.2%)	
3	0 (0.00%)	4 (5.88%)	
MDS-UPDRS Part I Anxious Mood			0.665
0	75 (65.2%)	42 (61.8%)	
1	37 (32.2%)	23 (33.8%)	
2	2 (1.74%)	3 (4.41%)	
3	1 (0.87%)	0 (0.00%)	
MDS-UPDRS Part I Apathy			0.369
0	100 (87.0%)	55 (80.9%)	
1	14 (12.2%)	13 (19.1%)	
2	1 (0.87%)	0 (0.00%)	
MDS-UPDRS Part I Depressed Mood			0.713
0	88 (76.5%)	49 (72.1%)	
1	24 (20.9%)	16 (23.5%)	
2	3 (2.61%)	3 (4.41%)	
MDS-UPDRS Part I Cognitive Impairment			0.414
0	88 (76.5%)	48 (70.6%)	
1	26 (22.6%)	18 (26.5%)	
2	1 (0.87%)	2 (2.94%)	
MDS-UPDRS Part I Hallucinations and Psychosis			1.000
0	110 (95.7%)	65 (95.6%)	
1	5 (4.35%)	3 (4.41%)	
MOCA Score (adjusted for education)	28.0 [26.5;29.0]	27.0 [25.0;29.0]	0.008*
UPSIT Score	23.0 [17.0;28.0]	20.0 [14.8;29.0]	0.385
Benton Judgement of Line Orientation Score	14.0 [12.5;15.0]	14.0 [12.0;15.0]	0.667
Epworth Sleepiness Scale Score	5.00 [3.00;6.50]	6.00 [4.00;9.00]	0.004*
REM SleepBehavior Disorder Questionnaire Score	3.00 [2.00;5.50]	3.00 [2.00;5.00]	0.794
Geriatric Depression Scale Score	2.00 [1.00;3.00]	2.00 [1.00;3.00]	0.211
STAI Total Score	60.0 [51.0;75.0]	64.0 [49.8;70.5]	0.990
Any QUIP disorder	28 (24.3%)	12 (17.6%)	0.266
SDMT Score	44.0 [37.0;50.0]	37.5 [32.8;45.0]	< 0.001*
HVLT Immediate/Total Recall	25.0 [22.0;30.0]	23.5 [20.8;26.0]	0.004*
HVLT Discrimination Recognition	10.0 [9.00;11.0]	10.0 [9.00;11.0]	0.282
HVLT Retention	0.90 [0.78;1.00]	0.88 [0.74;1.00]	0.247
SCOPA-AUT Gastrointestinal Score	1.00 [0.00;2.00]	2.00 [1.00;4.00]	0.008*
SCOPA-AUT Urinary Score	3.00 [2.00;5.00]	4.00 [2.00;6.00]	0.076
SCOPA-AUT Cardiovascular Score	0.00 [0.00;0.00]	0.00 [0.00;1.00]	0.085
SCOPA-AUT Thermoregulatory Score	1.00 [0.00;2.00]	1.00 [0.00;2.00]	0.781
SCOPA-AUT Pupillomotor Score	0.00 [0.00;3.00]	0.00 [0.00;2.00]	0.733
SCOPA-AUT Sexual Dysfunction Score	0.00 [0.00;2.00]	0.00 [0.00;2.00]	0.758
SCOPA-AUT Total Score	7.00 [4.50;11.0]	8.00 [6.00;13.2]	0.064
Semantic Fluency Total Score	50.0 [45.0;59.0]	47.0 [41.8;56.0]	0.053
Letter Number Sequencing Score	11.0 [9.00;13.0]	10.0 [9.00;12.0]	0.129
MCI test score (= 1)	0.10 (0.30)	0.19 (0.40)	0.087
Evolution of MDS UPDRS scores at year five (pre-FoG *n* = 68, non-FoG *n* = 115)			
Change of MDS-UPDRS Part I	3.00 [1.00;5.50]	5.00 [2.00;9.00]	0.004*
Change of MDS-UPDRS Part II	3.00 [1.00;6.00]	7.00 [3.00;11.0]	< 0.001*
Change of MDS-UPDRS Part III	9.00 [4.00;17.0]	13.5 [6.50;22.0]	0.066*

*P* <0.05 was presented with ‘*’

### Univariate analysis of FoG

**Fig. 1:**
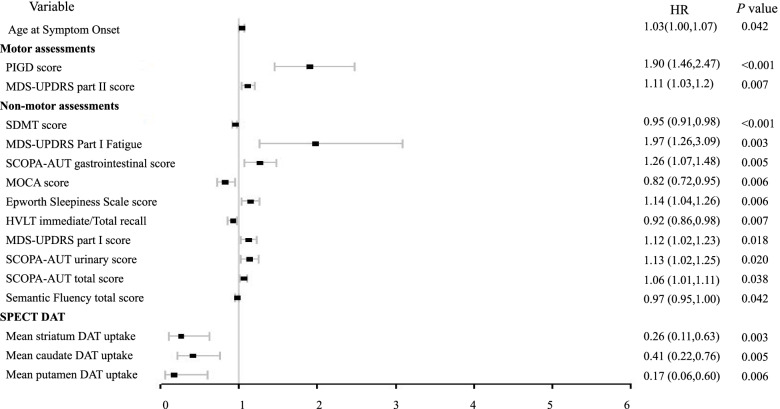
Univariate analysis of FoG onset during the 5-year follow up. Higher Age at symptom onset, higher PIGD score, higher MDS-UPDRS Part I & II score, fatigue, higher Epworth Sleepiness Scale score and autonomic dysfunctions were associated with FoG onset. PD patients with higher SDMT score, higher HVLT Immediate/Total Recall, higher MOCA score, higher Semantic Fluency total score and higher striatum DAT uptake were less likely to develop FoG within five years

Univariate logistic regression analysis showed that age at symptom onset, MDS-UPDRS part II score, TD and PIGD subtype could predict FoG occurrence. With regard to non-motor factors, cognitive tests including SDMT, HVLT Immediate/Total Recall and MOCA, non-cognitive tests including sleep disturbance: Epworth Sleepiness Scale, mood disorder: MDS-UPDRS Part I Fatigue and autonomic dysfunction: SCOPA-AUT gastrointestinal score, SCOPA-AUT urinary score and SCOPA-AUT total score were associated with FoG onset. (Table 3, Fig. 1).

**Table 3:** Univariate analysis of demographic, motor, non-motor and imaging parameters at baseline for FoG onset during the 5-year follow up

Index	OR	95% CI		*P*
**Demographic characteristics**				
Age (years)	1.03	1.00	1.06	0.064
Age at Symptom Onset	1.03	1.00	1.07	0.042*
Duration of Disease since Diagnosis (Months)	0.99	0.94	1.04	0.768
Gender, female	0.92	0.48	1.79	0.815
Years of education	1.01	0.91	1.11	0.892
Family members with PD (any)	0.86	0.55	1.32	0.485
Side most affected at PD onset	0.87	0.49	1.54	0.633
**Motor assessments**				
Categorical Hoehn & Yahr	1.18	0.65	2.13	0.580
Total rigidity score	0.98	0.87	1.10	0.677
TD/PIGD classification (TD)	0.33	0.17	0.64	0.001*
TD/PIGD classification (PIGD)	2.80	1.30	6.04	0.009*
TD/PIGD classification (Indeterminate)	2.03	0.84	4.89	0.116
PIGD score	1.90	1.46	2.47	< 0.001*
Tremor	0.94	0.85	1.04	0.241
MDS-UPDRS part II score	1.11	1.03	1.20	0.007*
MDS-UPDRS part III score	1.02	0.99	1.06	0.208
Modified Schwab & England ADL score	0.96	0.90	1.01	0.101
**Non-motor assessments**				
MOCA score	0.82	0.72	0.95	0.006*
MDS-UPDRS part I score	1.12	1.02	1.23	0.018*
MDS-UPDRS Part I Hallucinations and Psychosis	1.02	0.23	4.39	0.984
MDS-UPDRS Part I Apathy	1.42	0.66	3.07	0.373
MDS-UPDRS Part I Features of Dopamine Dysregulation Syndrome	1.71	0.24	12.4	0.595
MDS-UPDRS Part I Fatigue	1.97	1.26	3.09	0.003*
UPSIT score	0.99	0.95	1.02	0.445
Benton Judgement of Line Orientation score	0.96	0.83	1.11	0.581
REM sleep behavior disorder questionnaire score	1.00	0.89	1.12	0.973
Epworth Sleepiness Scale score	1.14	1.04	1.26	0.006*
Geriatric Depression Scale score	1.06	0.92	1.23	0.393
STAI total score	1.00	0.98	1.02	0.916
QUIP score	0.85	0.50	1.44	0.547
SDMT score	0.95	0.91	0.98	< 0.001*
HVLT Immediate/Total Recall	0.92	0.86	0.98	0.007*
SCOPA-AUT total score	1.06	1.01	1.11	0.030*
SCOPA-AUT cardiovascular score	1.39	0.92	2.10	0.113
SCOPA-AUT gastrointestinal score	1.26	1.07	1.48	0.005*
SCOPA-AUT pupillomotor score	0.76	0.45	1.27	0.290
SCOPA-AUT sexual dysfunction score	1.05	0.87	1.27	0.599
SCOPA-AUT thermoregulatory score	0.98	0.79	1.21	0.834
SCOPA-AUT urinary score	1.13	1.02	1.25	0.020*
Semantic Fluency total score	0.97	0.94	1.00	0.039*
Letter Number Sequencing score	0.90	0.80	1.01	0.067
MCI test score (= 1)	2.23	0.94	5.32	0.069
**SPECT-DAT**				
Mean caudate DAT uptake	0.41	0.22	0.76	0.005*
Mean putamen DAT uptake	0.17	0.06	0.60	0.006*
Mean striatum DAT uptake	0.26	0.11	0.63	0.003*

*P* <0.05 was presented with ‘*’

Reduction of DAT uptake in the striatum, both in the caudate and putamen, was a strong predictor of FoG occurrence (Table 3). Although the two groups did not differ significantly with regard to CSF biomarkers at baseline, univariate analysis identified CSF biomarker Abeta_42_ as a predictor of FoG onset (Table 4).

**Table 4:** Univariate analysis of CSF biomarkers and genetic pattern at baseline for FoG onset during the 5-year follow up

Index	OR	95% CI		*P*
**CSF biomarkers**				
Abeta	1.00	1.00	1.00	0.027*
Tau	1.00	0.99	1.00	0.622
pTau	1.00	0.94	1.06	0.885
aSyn	1.00	1.00	1.00	0.653
**Genetic Pattern**				
APOE	0.94	0.64	1.40	0.775
APOE Genotype—number of e4 alleles	0.87	0.48	1.60	0.663
SNCA_rs3910105	1.35	0.86	2.12	0.194
SNCA_rs356181	0.96	0.63	1.44	0.826
MAPT	0.96	0.57	1.61	0.865

In the regression analysis, different genetic features were represented as numbers as below:

APOE: e2/e4 ~ 1, e3/e2 ~ 2, e3/e3 ~ 3, e4/e3 ~ 4, e4/e4 ~ 5, e2/e2 ~ 6

SNCA_rs356181: C/C ~ 1, C/T ~ 2, T/T ~ 3

SNCA_rs3910105: C/C ~ 1, C/T ~ 2, T/T ~ 3

MAPT: H1/H1 ~ 1, H1/H2 ~ 2, H2/H2 ~ 3

*P* <0.05 was presented with ‘*’

Previous studies indicated that MDS-UPDRS evolution may predict FoG [4]. Thus, we investigated if MDS-UPDRS score changes detected at each annual visit relative to baseline levels correlated with FoG development. Over the course of 5 years, the correlation between the change of MDS-UPDRS score parameters and FoG increased. However, only the change of MDS-UPDRS part II score was significantly associated with FoG occurrence at each visit year (Table 5).

**Table 5:** Associations between the evolution of MDS UPDRS scores and FoG onset during the 5-year follow up

Index	OR	95% CI		*P*
Evolution of MDS UPDRS scores at year one (pre-FoG *n* = 64, non-FoG *n* = 112)				
Change of MDS-UPDRS part I	1.09	0.97	1.22	0.147
Change of MDS-UPDRS part II	1.09	1.00	1.18	0.050
Change of MDS-UPDRS part III	1.02	0.98	1.06	0.288
Evolution of MDS UPDRS scores at year two (pre-FoG *n* = 65, non-FoG *n* = 111)				
Change of MDS-UPDRS part I	1.18	1.07	1.30	0.001*
Change of MDS-UPDRS part II	1.22	1.10	1.34	< 0.001*
Change of MDS-UPDRS part III	1.03	0.98	1.07	0.221
Evolution of MDS UPDRS scores at year three (pre-FoG *n* = 68, non-FoG *n* = 111)				
Change of MDS-UPDRS part I	1.08	1.00	1.17	0.041
Change of MDS-UPDRS part II	1.11	1.03	1.20	0.004*
Change of MDS-UPDRS part III	1.03	1.00	1.06	0.084
Evolution of MDS UPDRS scores at year four (pre-FoG *n* = 65, non-FoG *n* = 112)				
Change of MDS-UPDRS part I	1.19	1.10	1.29	< 0.001*
Change of MDS-UPDRS part II	1.11	1.04	1.19	0.001*
Change of MDS-UPDRS part III	1.04	1.01	1.07	0.003*
Evolution of MDS UPDRS scores at year five (pre-FoG *n* = 69, non-FoG *n* = 116)				
Change of MDS-UPDRS part I	1.11	1.03	1.19	0.005*
Change of MDS-UPDRS part II	1.13	1.06	1.20	< 0.001*
Change of MDS-UPDRS part III	1.03	1.00	1.07	0.038*

*P* <0.05 was presented with ‘*’

### Predictive model of FoG

**Fig. 2:**
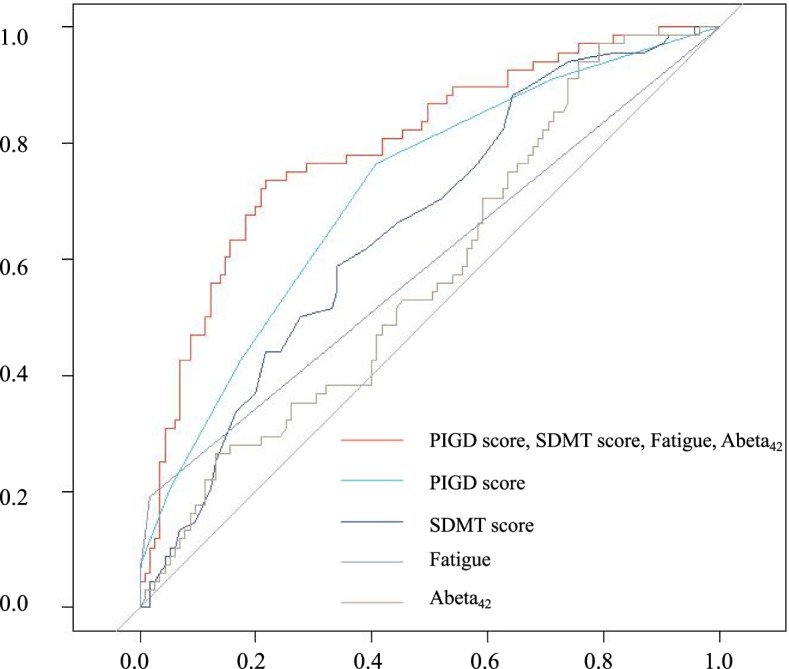
ROC curve analysis for the onset of FoG during the 5-year follow up. The AUC of the multivariate predictive model (TD, fatigue, SDMT score and Abeta) is 0.793

Next, we conducted the multivariate logistic regression analysis of factors that had *p* < 0.05 in the univariate anaysis using backward stepwise selection. As DAT imaging biomarkers (mean striatum, mean caudate, mean putamen) were highly related, only mean striatum DAT uptake value was entered in the analysis. This analysis identified PIGD score, MDS-UPDRS Part I Fatigue, SDMT score and Abeta_42_ as being strongly associated with FoG onset(Table 6). PD patients with higher PIGD score, higher MDS-UPDRS Part I Fatigue score, lower SDMT score, and lower CSF Abeta_42_ were at a higher risk of developing FoG. The AUC (area under curve) in the ROC analysis was 0.793 (Fig. 2, 95% CI: 0.725–0.861). The *p* value of the Hosmer and Lemeshow goodness-of-fit test was 0.496, indicating good calibrations. We applied our model derived from the complete-case analysis to the patient set that contained missing values. All variables were significantly associated with FoG occurrence in the univariate and multivariate analysis except MDS-UPDRS Part I Fatigue score which showed a marginal significance (*p* = 0.065*)* in the multivariate analysis. The AUC of the model was 0.761 (0.690–0.833).

**Table 6:** Multivariate analysis at baseline for the onset of FoG during the 5-year follow up

Index	OR	95% CI		*P*
PIGD score	1.78	1.36	2.39	< 0.001*
Abeta_42_	1.00	1.00	1.00	0.009*
SDMT score	0.96	0.92	0.99	0.013*
MDS-UPDRS Part I Fatigue	1.92	1.17	3.29	0.013*

Abeta OR 0.999, 95% CI: 0.998–1.000

*R*^2^ = 0.227 (Cox & Snell), *R*^2^ = 0.310 (Nagelkerke). Homer and Lemeshow Goodness of fitχ^2^ = 7.383, *p* = 0.496

*P* <0.05 was presented with ‘*’

To further exclude covariates, we adjusted our model for age, disease duration, and gender. However, as revealed by the multivariate model, none of these parameters correlated with FoG onset (Table S5). Therefore, we restricted our model to PIGD subscore, fatigue, SDMT score and Abeta_42_. The following equation represents the probability (p) of developing FoG within five years:

$$Log(p/1-p)=1.143+0.578\;(PIGD\;score)+0.654\;(MDS-UPDRS\;Part\;I\;Fatigue)-0.046\;(SDMT\;score)-0.001\;Abeta_{42}$$

## Discussion

In this study, we evaluated various aspects of potential risk factors to predict FoG onset in early developed PD patients. There were 183 patients enrolled in the study among whom 68 patients (37.2%) developed FoG in the follow up duration of five years. Overall, 31.1% of patients (57/183) reported ‘freezing when walking’ in their activities of daily living, while 17.5% of patients (32/183) were defined as FoG by the investigator in clinic (Table S1, Figure S1). The two groups showed good homogeneity in the baseline characteristics except for the differences in age (*p* = 0.03) and age at symptom onset (*p* = 0.05). Reasons for the differences observed are unknown but the small sample size may explain at least a part of it. The influence of the differences is likely to be limited as the two variables were excluded from the backward stepwise selection and age was adjusted as a covariate. The prevalence of FoG in the present study (37.2%) was comparable to previous studies, as a meta-analysis found that the weighted prevalence of FoG in early-stage PD patients with a disease duration ≤ 5 years was 37.9% [18]. The lower rate of FoG detected in clinic reflects the transient nature of FoG symptoms, suggesting a combination of questionnaire with regard to daily living can be more sensitive to reflect FoG development in PD patients.

Freezing of gait has been recognized as a neuronal integration failure caused by a multilevel brain network. It is influenced by cognitive, sensory-perceptual, and affective manipulations, and can be induced by several occasions such as turning, fatigue, confined spaces, and stressful situations [19, 20]. This study found that motor factors, along with non-motor factors including cognitive functions, mood and CSF Abeta predisposed PD patients to FoG development. While motor features have been recognized to correlate with FoG [4, 7], it was noticed that motor subtypes also correlated with non-motor features [21]. To further evaluate the difference in TD/PIGD subtype, we analyzed baseline features of different motor phenotypes (Table S2) in PD patients. No difference in Abeta, fatigue and SDMT scores was found significant at baseline between TD and PIGD subtypes. However, there were differences observed in education years, HVLT Retention, number of e4 alleles in APOE genotype and depression apart from the tremor score in groups. Although none of these factors were identified as risk factors in our studies, they may indirectly contribute to FoG through their influence on motor phenotypes. Previous studies have reported the associations between these factors and FoG. Therefor, we can not exclude their potential value in the prediction of FoG onset [10, 22]. As was shown by previous study conducted by Kim et al., PIGD score was a strong predictor of FoG [8]. In our analysis,the predictive power of PIGD score was stronger than that of TD or PIGD subtype. Besides, PIGD score also showed a correlation with fatigue and SDMT score in our analysis, supporting again the coexistence and inter-relationship of motor and non-motor symptoms in the development FoG.

Non-motor features are considered of increasing importance in the development of FoG [19]. In our study, fatigue and cognitive deficit (represented as lower SDMT scores), emerged as independent FoG predictors. In PD patients, fatigue is a major triggering factor of FoG [20]. It is associated with FoG occurrence in clinically observed FoG as well as self-reported FoG, despite late or early onset [23]. However, it is influenced by motor phenotype [21]. Noticeably, this study showed a correlation between PIGD score and fatigue (*r* = 0.22, *p* = 0.003, Table S3) at baseline. The control of PIGD symptoms and fatigue may be originiated from the same or adjacent neuronal circuit in FoG development while further investigations are warranted. Accumulating evidence has suggested cognitive effects, specifically, the executive functions, attention, and visuospatial functions to FoG occurrence [24]. In our study, SDMT, which is a commonly used instrument to evaluate cognitive functions especially attention deficit, was identified as an independent risk factor. While studies suggested visual and motor con-founders in SDMT interpretations [25], a recent study using gaze analysis technique excluded the confounding effects, further demonstrating the role of cognitive functions in SDMT performance in PD patients [26]. In this study, we demonstrate the role of SDMT performance in FoG prediction, which implicates therapeutics for cognitive rehabilitation, especially for attention improvement might help delay FoG onset in PD patients.

Low CSF Abeta_42_ levels are regarded as a biological fluid marker for Alzheimer's disease [27]. In this study, CSF Abeta_42_ also correlated with FoG in PD patients. The decreased levels of CSF Abeta are associated with cognitive impairments and gait symptoms in PD [27, 28]. Extra-nigral pathologies, represented as the increased neocortical Abeta deposition, can significantly increase the risk of FoG development [29]. However, it remains unclear how the reduced level of CSF Abeta_42,_ or the deposition of Abeta_42_ contributes to the motor dysregulation in PD patients [29].

Our study has several limitations. First, it is a retrospective study with a limited number of subjects in the PPMI study. Participants in PPMI were generally well educated, which may not be representative of the other group. Second, we could not distinguish the medication states of patients identified by MDS-UPDRS Part II, thus restricting our study of FoG under different medication conditions. Third, we did not analyze the severity of FoG. The combination of UPDRS II and UPDRS III for FoG identification made it difficult to find a consistent standard to measure the severity. Besides, UPDRS II may be less sensitive to detect FoG compared with the FoG questionnaire (FOG-Q) [30]. The number of freezers may still be underestimated in the analysis. Fourth, we built our model from the complete-case analysis. Data with missing values were deleted and this may introduce selection bias. The difference in results was indeed observed. As in the multivariate logistic regression analysis on 251 patients, variables that were selected in the model were PIGD score, SCOPA-AUT gastrointestinal (GI) score, SDMT score, mean striatum DAT uptake and Abeta. Nevertheless, all the variables in our model were significantly associated with FoG onset in the patient set with missing values, showing good consistency in the results. In this study, we evaluated FoG with both self-reported rating scales and examinations from clinical specialists. We integrated a comprehensive battery of clinical, biochemical, and imaging assessments and emphasized several independent risk factors. Future prospective studies integrating the identified factors under different medication status may further demonstrate their prognostic value and deepen our understanding of the development of FoG in PD patients.

## Conclusions

In summary, our findings determined the risk factors of FoG occurrence among a series of clinical, imaging, biological as well as genetic characteristics. Our results stress the importance of PIGD score, fatigue, SDMT performance and CSF Abeta_42_ in predicting FoG onset. Combining these factors with further studies will assist patients, caregivers, and healthcare professionals to conduct early interventions as disease progresses. Physiotherapy, pharmacological treatments, or neuromodulation that improve the performance of these indicators will be of value in the early intervention of the debilitating symptom.

## Supplementary Information

**Additional file 1.**

## Data Availability

The data that support the findings of this study are available from PPMI website (www.ppmi-info.org/data). Restrictions apply to the availability of these data, which were used under license for the current study, and so are not publicly available. Data are however available from the authors upon reasonable request and with permission of PPMI.

## Abbreviations

PD: Parkinson’s disease
PPMI: The Parkinson’s Progression Markers Initiative
FoG: Freezing of gait
TD: Tremor dominant
PIGD: Postural instability/gait difficulty
ROC: Receiver operating characteristic
DAT: Dopamine transporter
SDMT: Symbol Digit Modalities Test
HVLT: Hopkins Verbal Learning Test
MOCA: Montreal Cognitive Assessment
SCOPA-AUT: Scales for Outcomes in Parkinson's Disease‐Autonomic
MDS: Movement Disorders Society
UPDRS: Unified Parkinson’s disease rating scale
MCI: Mild cognitive impairment
UPSIT: University of Pennsylvania Smell Identification Test
QUIP: Questionnaire for Impulsive‐Compulsive Disorders
CSF: Cerebrospinal fluid
STN: Subthalamic nucleus
GPi: Globus pallidus internus
SNr: Substantia nigra pars reticulata
SPECT: Single photon emission computed tomography
APOE: Apolipoprotein
MAPT: Microtubule-associated protein tau
SNCA: α-Synuclein
GCP: Good clinical practices
