# Supplementary material for: Predicting the onset of freezing of gait in Parkinson’s disease
Source: BMC Neurol. 2022 Jun 7;22:213. doi: 10.1186/s12883-022-02713-2 (PMC9172010; doi:10.1186/s12883-022-02713-2)

**Table S1** Number of FoG patients identified by MDS-UPDRS2 or MDS-UPDRS3.

| **Visit Year** | ***n* (MDS-UPDRS2)** | ***n* (MDS-UPDRS3)** | ***n* (BOTH)** | ***n* (MDS-UPDRS3 DOSE ON)** | ***n* (MDS-UPDRS3 DOSE OFF)** | ***n***  **(IN TOTAL)** |
| --- | --- | --- | --- | --- | --- | --- |
| **1** | 16 | 7 | 1 | 0 | 7 | 22 |
| **2** | 27 | 12 | 3 | 2 | 10 | 36 |
| **3** | 32 | 18 | 7 | 6 | 14 | 43 |
| **4** | 44 | 21 | 8 | 7 | 18 | 57 |
| **5** | 57 | 32 | 21 | 15 | 23 | 68 |

**Figure S1** Number of FoG patients identified by MDS-UPDRS2 or MDS-UPDRS3.


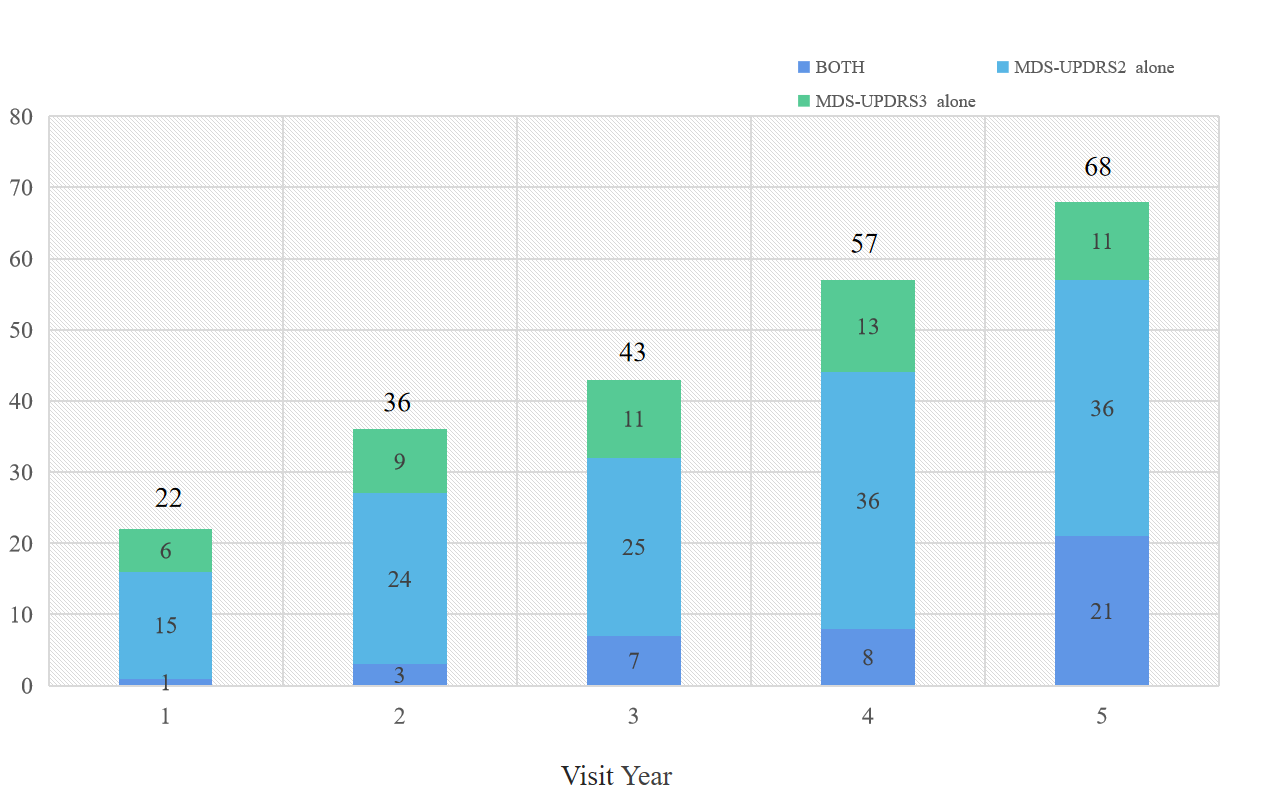


**Table S2** Baseline characteristics of TD, PIGD and indeterminate patients.

|  | **TD**  **(*n*=127)** | **PIGD**  **(*n*=33)** | **Indeterminate**  **(*n*=23)** | ***P***  **(TD VS PIGD)** | | 1. **Overall** | | ***P*** | |  |
| --- | --- | --- | --- | --- | --- | --- | --- | --- | --- | --- |
| **Demographic information** |  |  |  |  | |  | |  | |  |
| Age (years) | 62.0 [54.7;68.9] | 61.4 [53.1;68.5] | 59.6 [51.2;69.7] | 0.868 | | 0.632 | |  | |  |
| Age at Symptom Onset (years) | 60.3 [52.6;66.4] | 60.1 [52.0;66.8] | 58.6 [49.3;69.2] | 0.994 | | 0.958 | |  | |  |
| Duration of Disease since Diagnosis (Months) | 4.23 [2.18;7.70] | 3.30 [2.30;5.67] | 4.80 [2.77;6.17] | 0.469 | | 0.319 | |  | |  |
| Gender, female | 38 (29.9%) | 9 (27.3%) | 6 (26.1%) | 0.907 | | 0.934 | |  | |  |
| Years of education | 16.0 [15.0;18.0] | 14.0 [12.0;18.0] | 16.0 [14.0;17.5] | 0.033* | | 0.015* | |  | |  |
| Categorical Education |  |  |  | 0.002* | | <0.001* | |  | |  |
| <13 yrs | 13 (10.2%) | 12 (36.4%) | 4 (17.4%) |  | |  | |  | |  |
| 13-23 yrs | 113 (89.0%) | 20 (60.6%) | 19 (82.6%) |  | |  | |  | |  |
| >23 yrs | 1 (0.79%) | 1 (3.03%) | 0 (0.00%) |  | |  | |  | |  |
| Family members with PD (any) | 27 (21.3%) | 12 (36.4%) | 7 (30.4%) | 0.168 | | 0.116 | |  | |  |
| **Disease characteristics** |  |  |  |  | |  | |  | |  |
| Side most affected at PD onset |  |  |  | 0.411 | | 0.331 | |  | |  |
| Left | 55 (43.3%) | 16 (48.5%) | 11 (47.8%) |  | |  | |  | |  |
| Right | 71 (55.9%) | 16 (48.5%) | 11 (47.8%) |  | |  | |  | |  |
| Symmetric | 1 (0.79%) | 1 (3.03%) | 1 (4.35%) |  | |  | |  | |  |
| **SPECT-DAT** |  |  |  |  | |  | |  | |  |
| Mean caudate DAT uptake | 2.10 (0.51) | 1.93 (0.56) | 1.90 (0.44) | 0.096 | | 0.126 | |  | |  |
| Mean putamen DAT uptake | 0.84 [0.69;0.99] | 0.74 [0.62;1.00] | 0.72 [0.59;0.84] | 0.179 | | 0.334 | |  | |  |
| Mean striatum DAT uptake | 1.47 (0.36) | 1.37 (0.40) | 1.35 (0.38) | 0.185 | | 0.182 | |  | |  |
| **CSF biomarkers** |  |  |  |  | |  | |  | |  |
| Abeta | 893 [692;1140] | 961 [695;1251] | 898 [658;1174] | 0.814 | | 0.569 | |  | |  |
| aSyn | 1468 [1121;1798] | 1459 [1160;1866] | 1310 [1049;1539] | 0.415 | | 0.918 | |  | |  |
| Tau | 161 [138;209] | 172 [142;210] | 160 [128;182] | 0.541 | | 0.795 | |  | |  |
| pTau | 13.6 [11.2;17.2] | 14.2 [11.4;17.7] | 12.9 [10.7;15.5] | 0.588 | | 0.711 | |  | |  |
| **Genetic Pattern** |  |  |  |  | |  | |  | |  |
| APOE |  |  |  | 0.434 | | 0.520 | |  | |  |
| APOE Genotype - number of e4 alleles | 0.31 (0.51) | 0.15 (0.36) | 0.43 (0.59) | 0.100 | | 0.040* | |  | |  |
| SNCA_rs356181 |  |  |  | 0.276 | | 0.081 | |  | |  |
| SNCA_rs3910105 |  |  |  | 0.072 | | 0.079 | |  | |  |
| MAPT |  |  |  | 0.113 | | 0.403 | |  | |  |
| **Motor assessments** |  |  |  |  | |  | |  | |  |
| Categorical Hoehn & Yahr |  |  |  | 0.149 | | 0.236 | |  | |  |
| Stage 1 | 61 (48.0%) | 16 (48.5%) | 7 (30.4%) |  | |  | |  | |  |
| Stage 2 | 66 (52.0%) | 16 (48.5%) | 16 (69.6%) |  | |  | |  | |  |
| Stages 3-5 | 0 (0.00%) | 1 (3.03%) | 0 (0.00%) |  | |  | |  | |  |
| Total Rigidity Score | 3.00 [2.00;5.00] | 4.00 [2.00;5.00] | 4.00 [2.50;5.00] | 0.694 | | 0.488 | |  | |  |
| Tremor Score | 5.00 [3.00;7.00] | 0.00 [0.00;1.00] | 1.00 [1.00;3.50] | <0.001* | | <0.001* | |  | |  |
| Modified Schwab & England ADL Score | 95.0 [90.0;100] | 90.0 [90.0;100] | 90.0 [90.0;100] | 0.471 | | 0.778 | |  | |  |
| MDS-UPDRS Part II Score | 4.00 [2.00;7.00] | 6.00 [3.00;9.00] | 7.00 [4.00;11.5] | 0.013* | | 0.145 | |  | |  |
| MDS-UPDRS Part III Score | 20.0 [14.0;24.5] | 20.0 [14.0;26.0] | 19.0 [11.5;24.5] | 0.778 | | 0.798 | |  | |  |
| MDS-UPDRS Total Score | 29.0 [22.0;37.5] | 30.0 [21.0;38.0] | 31.0 [21.0;41.5] | 0.825 | | 0.775 | |  | |  |
| **Non-motor assessments** |  |  |  |  | |  | |  | |  |
| MDS-UPDRS Part I Score | 5.00 [2.00;7.00] | 5.00 [2.00;7.00] | 6.00 [3.00;7.00] | 0.667 | | 0.835 | |  | |  |
| MDS-UPDRS Part I Features of Dopamine Dysregulation Syndrome |  |  |  | 0.757 | | 0.057 | |  | |  |
| 0 | 123 (96.9%) | 33 (100%) | 23 (100%) |  | |  | |  | |  |
| 1 | 4 (3.15%) | 0 (0.00%) | 0 (0.00%) |  | |  | |  | |  |
| MDS-UPDRS Part I Fatigue |  |  |  | 0.044* | | 0.175 | |  | |  |
| 0 | 72 (56.7%) | 15 (45.5%) | 8 (34.8%) |  | |  | |  | |  |
| 1 | 48 (37.8%) | 15 (45.5%) | 10 (43.5%) |  | |  | |  | |  |
| 2 | 6 (4.72%) | 1 (3.03%) | 4 (17.4%) |  | |  | |  | |  |
| 3 | 1 (0.79%) | 2 (6.06%) | 1 (4.35%) |  | |  | |  | |  |
| MDS-UPDRS Part I Anxious Mood |  |  |  | 0.822 | | 0.892 | |  | |  |
| 0 | 81 (63.8%) | 23 (69.7%) | 13 (56.5%) |  | |  | |  | |  |
| 1 | 41 (32.3%) | 9 (27.3%) | 10 (43.5%) |  | |  | |  | |  |
| 2 | 4 (3.15%) | 1 (3.03%) | 0 (0.00%) |  | |  | |  | |  |
| 3 | 1 (0.79%) | 0 (0.00%) | 0 (0.00%) |  | |  | |  | |  |
| MDS-UPDRS Part I Apathy |  |  |  | 0.048* | | 0.385 | |  | |  |
| 0 | 112 (88.2%) | 27 (81.8%) | 16 (69.6%) |  | |  | |  | |  |
| 1 | 15 (11.8%) | 6 (18.2%) | 6 (26.1%) |  | |  | |  | |  |
| 2 | 0 (0.00%) | 0 (0.00%) | 1 (4.35%) |  | |  | |  | |  |
| MDS-UPDRS Part I Depressed Mood |  |  |  | 0.066 | | 0.057 | |  | |  |
| 0 | 100 (78.7%) | 20 (60.6%) | 17 (73.9%) |  | |  | |  | |  |
| 1 | 25 (19.7%) | 11 (33.3%) | 4 (17.4%) |  | |  | |  | |  |
| 2 | 2 (1.57%) | 2 (6.06%) | 2 (8.70%) |  | |  | |  | |  |
| MDS-UPDRS Part I Cognitive Impairment |  |  |  | 0.203 | | 0.178 | |  | |  |
| 0 | 97 (76.4%) | 24 (72.7%) | 15 (65.2%) |  | |  | |  | |  |
| 1 | 29 (22.8%) | 7 (21.2%) | 8 (34.8%) |  | |  | |  | |  |
| 2 | 1 (0.79%) | 2 (6.06%) | 0 (0.00%) |  | |  | |  | |  |
| MDS-UPDRS Part I Hallucinations and Psychosis |  |  |  | 1.000 | | 1.000 | |  | |  |
| 0 | 121 (95.3%) | 32 (97.0%) | 22 (95.7%) |  | |  | |  | |  |
| 1 | 6 (4.72%) | 1 (3.03%) | 1 (4.35%) |  | |  | |  | |  |
| MOCA Score (adjusted for education) | 28.0 [26.0;29.0] | 28.0 [26.0;28.0] | 28.0 [27.0;29.0] | 0.889 | | 0.778 | |  | |  |
| UPSIT Score | 22.0 [15.0;27.0] | 23.0 [17.0;31.0] | 22.0 [15.0;28.5] | 0.660 | | 0.446 | |  | |  |
| Benton Judgement of Line Orientation Score | 14.0 [13.0;15.0] | 13.0 [12.0;14.0] | 14.0 [13.0;15.0] | 0.158 | | 0.084 | |  | |  |
| Epworth Sleepiness Scale Score | 5.00 [3.00;7.00] | 6.00 [4.00;9.00] | 8.00 [4.00;8.50] | 0.074 | | 0.098 | |  | |  |
| REM Sleep Behavior Disorder Questionnaire Score | 3.00 [2.00;5.00] | 3.00 [2.00;6.00] | 3.00 [2.00;5.00] | 0.825 | | 0.556 | |  | |  |
| Geriatric Depression Scale Score | 1.00 [1.00;2.00] | 3.00 [2.00;3.00] | 2.00 [1.00;3.50] | 0.006* | | 0.002* | |  | |  |
| STAI Total Score | 58.0 [50.0;71.5] | 65.0 [52.0;78.0] | 68.0 [60.0;79.0] | 0.119 | | 0.243 | |  | |  |
| Any QUIP disorder | 31(24.4%) | 4(12.1%) | 5(21.7%) | 0.651 | | 0.412 | |  | |  |
| SDMT Score | 42.0 [34.0;48.0] | 42.0 [32.0;46.0] | 43.0 [36.0;49.5] | 0.653 | | 0.734 | |  | |  |
| HVLT Immediate/Total Recall | 25.0 [21.0;28.5] | 25.0 [21.0;28.0] | 25.0 [22.5;27.5] | 0.955 | | 0.848 | |  | |  |
| HVLT Discrimination Recognition | 10.0 [9.00;11.0] | 10.0 [9.00;11.0] | 10.0 [9.50;11.0] | 0.701 | | 0.447 | |  | |  |
| HVLT Retention | 0.90 [0.79;1.00] | 0.80 [0.67;1.00] | 0.83 [0.73;1.00] | 0.022* | | 0.009* | |  | |  |
| SCOPA-AUT Total Score | 7.00 [5.00;11.0] | 6.00 [5.00;12.0] | 8.00 [7.50;14.0] | 0.181 | | 0.604 | |  | |  |
| Semantic Fluency Total Score | 48.0 [42.0;57.5] | 49.0 [44.0;62.0] | 49.0 [45.0;58.0] | 0.638 | | 0.530 | |  | |  |
| Letter Number Sequencing Score | 11.0 [9.00;13.0] | 11.0 [9.00;13.0] | 11.0 [9.00;12.0] | 0.892 | | 0.914 | |  | |  |
| MCI test score (= 1) | 0.12 (0.32) | 0.18 (0.39) | 0.13 (0.34) |  | 0.631 | 0.394 |  | |  | |

**Table S3** Correlations of PIGD score, SDMT score, fatigue and CSF Abeta in PD patients.

|  |  | PIGD score | Fatigue | SDMT score | Abeta |
| --- | --- | --- | --- | --- | --- |
| PIGD score | Pearson's r | 1 | 0.221 | -0.201 | 0.041 |
|  | *P* |  | 0.003* | 0.006* | 0.580 |
| Fatigue | Pearson's r | 0.221 | 1 | 0.019 | 0.077 |
|  | *P* | 0.003* |  | 0.795 | 0.299 |
| SDMT score | Pearson's r | -0.201 | 0.019 | 1 | 0.016 |
|  | *P* | 0.006* | 0.795 |  | 0.826 |
| Abeta | Pearson's r | -0.018 | 0.077 | 0.016 | 1 |
|  | *P* | 0.808 | 0.299 | 0.826 |  |

**Table S4** Percentage of missing data

|  | Total Number | Missing  Number | Percentage（%) |
| --- | --- | --- | --- |
| bjlot | 250 | 1 | 0.4 |
| fampd_new | 250 | 1 | 0.4 |
| hvlt_immediaterecall | 250 | 1 | 0.4 |
| hvlt_discrimination | 249 | 2 | 0.8 |
| hvlt_retention | 250 | 1 | 0.4 |
| lns | 250 | 1 | 0.4 |
| scopa_gi | 250 | 1 | 0.4 |
| scopa_sex | 249 | 2 | 0.8 |
| scopa | 248 | 3 | 1.2 |
| VLTANIM | 250 | 1 | 0.4 |
| VLTVEG | 250 | 1 | 0.4 |
| VLTFRUIT | 250 | 1 | 0.4 |
| sft | 250 | 1 | 0.4 |
| SDMTOTAL | 250 | 1 | 0.4 |
| abeta | 244 | 7 | 2.9 |
| asyn | 246 | 5 | 2 |
| tau | 239 | 12 | 4.8 |
| ptau | 226 | 25 | 10 |
| mean_caudate | 248 | 3 | 1.2 |
| mean_putamen | 248 | 3 | 1.2 |
| mean_striatum | 248 | 3 | 1.2 |
| APOE | 224 | 27 | 12.1 |
| SNCA_rs356181 | 230 | 21 | 18.4 |
| SNCA_rs3910105 | 230 | 21 | 18.4 |
| APOE_e4 | 224 | 27 | 12.1 |
| MAPT | 230 | 21 | 9.1 |

**Table S5** Multivariate analysis adjusted for age, disease duration and gender for the onset of FoG during the 5-year follow up.

| **Index** | **OR** | **95% CI:** | | ***P*** |
| --- | --- | --- | --- | --- |
| PIGD score | 1.83 | 1.38 | 2.48 | <0.001* |
| MDS-UPDRS Part I Fatigue | 1.99 | 1.20 | 3.43 | 0.010* |
| SDMT score | 0.95 | 0.91 | 0.98 | 0.008* |
| Abeta | 1.00 | 1.00 | 1.00 | 0.008* |
| Age | 0.99 | 0.95 | 1.03 | 0.520 |
| Duration of Disease since Diagnosis (Months) | 0.98 | 0.92 | 1.04 | 0.528 |
| Gender | 1.50 | 0.68 | 3.33 | 0.320 |

Abeta: OR 0.999, 95% CI: 0.998-1.000.
*R*^2^ = 0.234 (Cox & Snell), *R*^2^ =0.320 (Nagelkerke). Homer and Lemeshow Goodness of fitχ^2^ = 5.812, *p=*0.668.

**Figure S2** ROC curve analysis for the onset of FoG during the 5-year follow up adjusted for age, gender and disease duration.


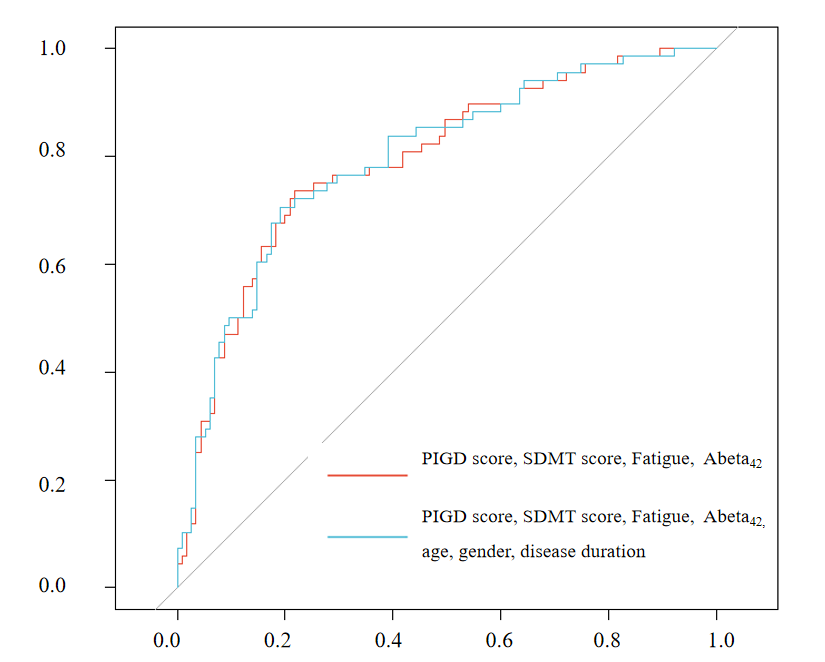

Supplement: Supplementary file 1 — Additional file 1. [file 12883_2022_2713_MOESM1_ESM.docx]
